# Normal serum alanine aminotransferase and non-alcoholic fatty liver disease among Korean adolescents: a cross-sectional study using data from KNHANES 2010–2015

**DOI:** 10.1186/s12887-018-1202-z

**Published:** 2018-07-05

**Authors:** Yunkoo Kang, Sowon Park, Seung Kim, Hong Koh

**Affiliations:** Department of Pediatrics, Severance Children’s Hospital, Severance Pediatric Liver Disease Research Group, Yonsei University College of Medicine, 50-1 Yonsei-ro, Seodaemun-gu, Seoul, 03722 Republic of Korea

**Keywords:** Non-alcoholic fatty liver disease, Alanine aminotransferase, Korea, Upper normal limit

## Abstract

**Background:**

Non-alcoholic fatty liver disease (NAFLD) is complicated disease and increasing worldwide. Previously, many studies of NALFD prevalences have used alanine aminotransferase (ALT) of > 40 U/L to define NAFLD, although that is too high to be reliable among adolescents. This study aimed to define the upper normal limit of ALT among Korean adolescents, and use it to estimate the prevalence of NAFLD, based on data from the Korea National Health and Nutrition Examination Survey (KNHANES).

**Methods:**

Data were obtained from 1785 healthy adolescents (916 boys and 869 girls, 10–18 years old) who participated in the KNHANES during 2010–2015. The International Diabetes Federation metabolic syndrome criteria for adolescents were used to exclude participants with metabolic syndrome components. Furthermore, participants who previously had diseases related to low HDL levels, high TG levels, diabetes, or very low/high body mass index and hepatitis B were excluded. The 95th percentiles level of ALT from healthy participants were evaluated. The definition of NAFLD was overweight status (≥85th percentile of body mass index) plus elevated ALT levels (95th percentile).

**Results:**

The upper normal ALT were 24.1 U/L for boys and 17.7 U/L for girls. Based on these values, the estimated prevalences of NAFLD in 2015 were 8.9% among adolescents.

**Conclusion:**

Defining the upper normal limit of ALT can be adjusted for each sex and ethnics in the general population. ALT laboratory thresholds used for children should be re-examined. The physicians should be aware not to underdiagnose NAFLD patient even ALT level is < 40 U/L.

## Background

### General and specific background

The prevalence of non-alcoholic fatty liver disease (NAFLD) is increasing worldwide. [1] Therefore, it is necessary for obese adolescents to be actively examined for NAFLD. And symptoms and clinical signs, laboratory and radiological imaging test, and liver biopsy is needed to make diagnosis finally as NAFLD. [2, 3] But in general population, it is not possible to perform all diagnostic procedures for each people. Therefore, Alanine aminotransferase (ALT) is used to find prevalence in general populations. ALT is an enzyme that is found in the cytosol of hepatocytes, and blood levels of ALT increase after liver injury [4]. Thus, blood testing for ALT is used globally as a minimally invasive and inexpensive tool for detecting chronic liver diseases, such as non-alcoholic fatty liver disease (NAFLD) [1].

### Debating issue

However, the reference ranges for normal ALT vary widely across different laboratories and populations [1]. Among adults, the upper normal limit of ALT was derived from healthy Italian blood donors, with values of 30 U/L for men and 19 U/L for women [5]. Among American adolescents, the upper normal limit of ALT was estimated by the National Health and Nutrition Examination Survey to be 25.8 U/L for boys and 22.1 U/L for girls [6, 7].

### Specific purpose of this study

Nevertheless, population differences indicate that these values may not be the same among Korean adolescents. However, the method used to define NAFLD in this study is only tools to estimated NAFLD in public populations, so this result cannot directly be used in clinic. But result of this study will give clues to evaluate and manage NAFLD in clinic. Therefore, the present study aimed to estimate the upper normal limits of ALT among Korean adolescents, as well as the prevalences of NAFLD based on those values.

## Methods

### Database

The present study evaluated data from the 2010–2015 Korea National Health and Nutrition Examination Survey (KNHANES). These annual cross-sectional surveys are performed using multi-stage probability samples that are representative of the general Korean population. The data of KNHANES surveys are available at http://knhanes.cdc.go.kr/.

### Study sample.

**Fig. 1:**
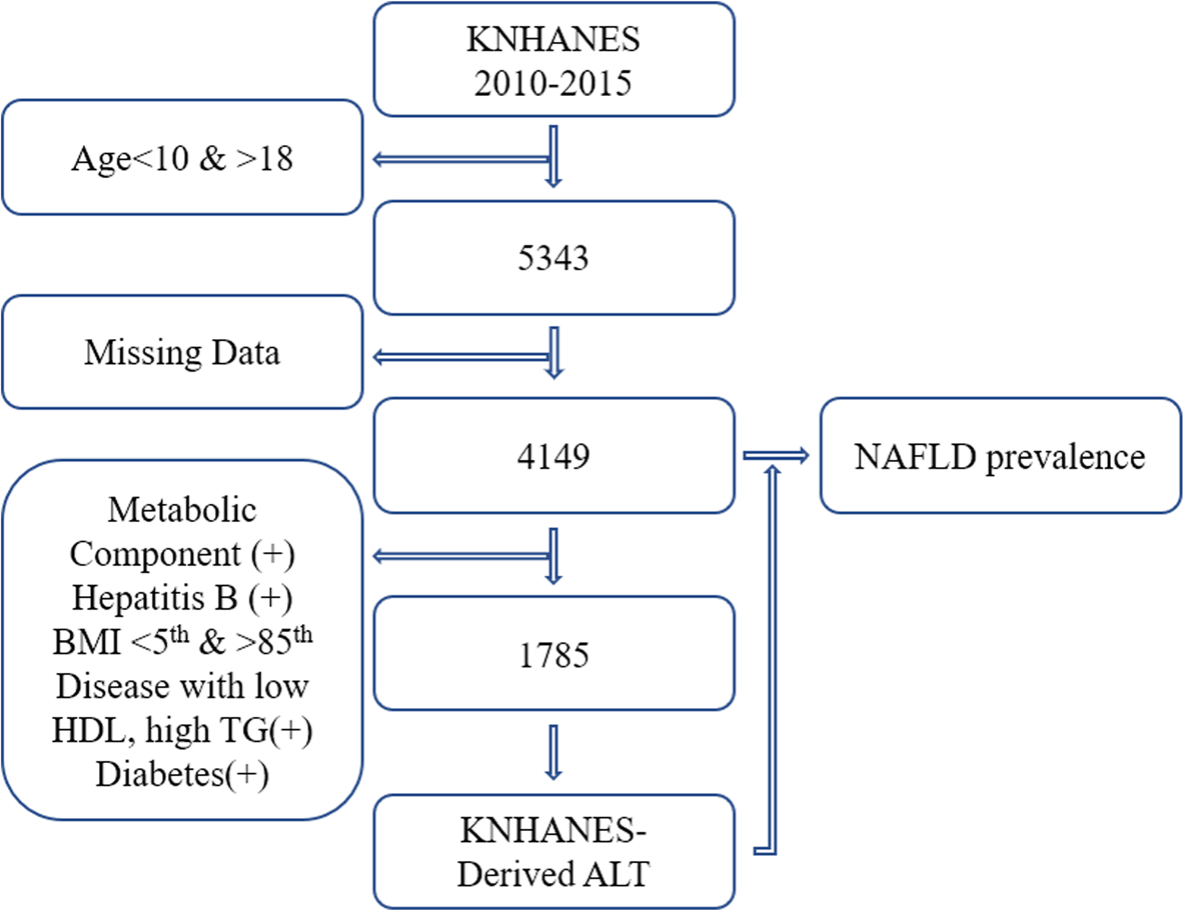
Flow chart for participant selection (916 boys, 869 girls)

During 2010–2015, 48,482 individuals participated in the KNHANES. The present study included participants who were 10–18 years old, but excluded participants with missing data and metabolic syndrome components, based on the International Diabetes Federation consensus definition (www.idf.org). Furthermore, 10–15-year-old participants were excluded if they had a waist circumference of ≥90th percentile for sex and age, triglyceride (TG) levels of ≥150 mg/dL, high density lipoprotein (HDL) levels of < 40 mg/dL, systolic blood pressure (SBP) of ≥130 mmHg, diastolic blood pressure (DBP) of ≥85 mmHg, and glucose levels of ≥100 mg/dL. Moreover, participants who were 16–18-years-old were excluded if they had a waist circumference of ≥90 cm or HDL levels of < 40 mg/dL (boys), a waist circumference of ≥80 cm or HDL levels of < 50 mg/dL (girls), TG levels of ≥150 mg/dL, SBP of ≥130 mmHg, DBP of ≥85 mmHg, and glucose levels of ≥100 mg/dL. Finally, we excluded participants who previously had diseases related to low HDL levels, high TG levels, diabetes, or very low/high body mass index (BMI, <5th percentile or > 85th percentile) [8]. Furthermore, participants who had hepatitis B infection were excluded. Thus, 1785 healthy participants (916 boys and 869 girls) were included in the analysis to determine the upper normal limit of ALT. For the present study, NAFLD was defined as being overweight (≥85th percentile of BMI) plus having elevated ALT (≥95th percentile, 24.1 U/L for boys and 17.7 U/L for girls). A total of 4149 participants (2226 boys and 1923 girls) were included in the analyses to estimate the prevalence of NAFLD (Figure 1) [9].

### Statistical analysis

All statistical analyses were performed using SPSS software (version 23.0; IBM Inc., Armonk, NY). Categorical data were collected for healthy participants (without metabolic syndrome components), and continuous laboratory and anthropometry data were expressed as mean ± standard error. The 95th percentiles for ALT were estimated for each sex using weighted analysis, and those values were used to estimate the prevalences of NAFLD among adolescents who participated in KNHANES and had available BMI and ALT data.

## Results

### Characteristics of the healthy participants

**Fig. 2:**
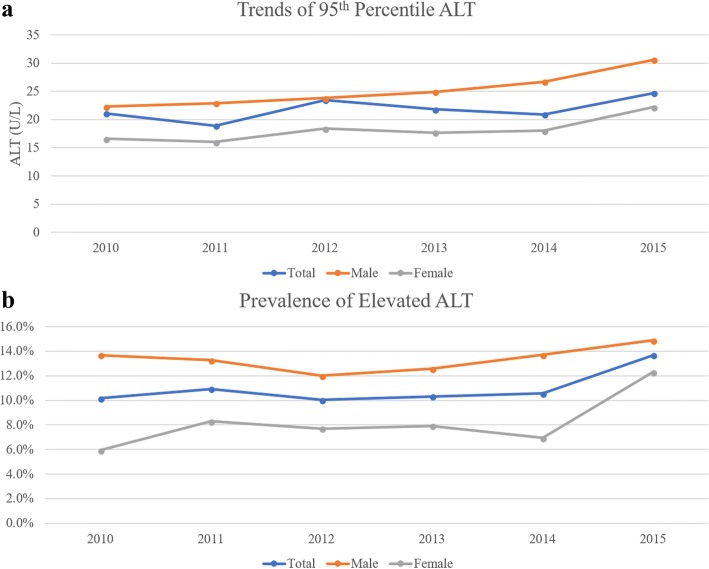
Trends in the 95th percentiles for alanine aminotransferase (**a**) and the prevalence of participants with elevated alanine aminotransferase (>95th percentile) (**b**)

The characteristics of the 1785 healthy participants (916 boys and 869 girls) are shown in Table 1. All characteristics appeared to be within the normal ranges, and the mean ALT levels were 14.2 U/L for boys and 10.9 U/L for girls. The 95th percentiles for ALT among healthy participants were 24.1 U/L for boys and 17.7 U/L for girls (Figure 2a).

**Table 1:** The characteristics of healthy KNHANES participants during 2010–2015

	Total	Male	Female
Age(year)	13.98 ± 0.07	13.98 ± 0.10	13.99 ± 0.10
SBP(mmHg)	105.39 ± 0.28	107.01 ± 0.41	103.78 ± 0.35
DBP(mmHg)	64.99 ± 0.27	64.98 ± 0.41	65.00 ± 0.32
Height(cm)	160.12 ± 0.30	163.54 ± 0.50	156.70 ± 0.31
Weight(kg)	50.35 ± 0.29	53.06 ± 0.49	47.63 ± 0.30
Waist(cm)	66.24 ± 0.18	67.77 ± 0.27	64.70 ± 0.22
BMI(kg/m^2^)	19.41 ± 0.06	19.56 ± 0.10	19.26 ± 0.07
Glucose(mg/dl)	88.52 ± 0.17	89.00 ± 0.22	88.04 ± 0.22
Cholesterol(mg/dl)	158.80 ± 0.74	152.82 ± 1.01	164.79 ± 1.06
HDL(mg/dl)	55.06 ± 0.29	53.54 ± 0.39	56.57 ± 0.40
TG(mg/dl)	68.76 ± 0.78	66.42 ± 1.09	71.11 ± 1.07
AST(U/L)	18.60 ± 0.15	20.02 ± 0.22	17.18 ± 0.17
ALT(U/L)	12.56 ± 0.18	14.18 ± 0.30	10.94 ± 0.19

Data are presented as mean ± standard error

*SBP* Systolic blood pressure, *DBP* Diastolic blood pressure, *BMI* Body mass index, *HDL* High-density lipoprotein, *TG* Triglycerides, *AST* Aspartate aminotransferase, *ALT* Alanine aminotransferase

### Prevalence of elevated ALT among Korean adolescents

A total of 4149 KNHANES participants had available ALT and BMI data. Among all participants during 2010–2015, the estimated prevalence of elevated ALT levels was 10.9% (95% confidence interval [CI]: 9.8–12.1%). During 2015, the overall estimated prevalence of elevated ALT levels was 13.7% (95% CI: 11.0–15.5%). In addition, the estimated prevalences of elevated ALT levels during 2015 were 14.9% (95% CI: 11.0–19.8%) for boys and 12.3% (95% CI: 8.6–17.3%) for girls (Figure 2b).

### Prevalence of NAFLD among Korean adolescents

**Fig. 3:**
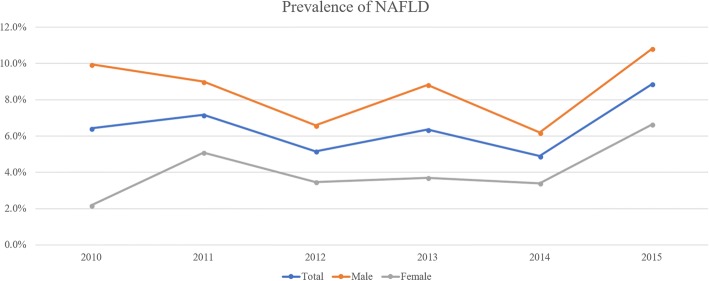
The estimated prevalence of non-alcoholic fatty liver disease among Korean adolescents

The prevalence of NAFLD was estimated using the 95th percentile values for ALT (24.1 U/L for boys and 17.7 U/L for girls) plus the age- and sex-specific 85th percentile values for BMI. During 2015, the overall prevalence of NAFLD was 8.9% (95% CI: 6.7–11.6%), with prevalences of 10.8% (95% CI: 7.7–15.0%) among boys and 6.6% (95% CI: 4.0–10.9%) among girls (Figure 3).

## Discussion

The present study evaluated data from the general population of healthy Korean adolescents to estimate the upper normal limit of ALT. The results indicate that the upper limits (95th percentiles) for estimating the prevalence of NAFLD in this population were 24.1 U/L for boys and 17.7 U/L for girls. These values are similar to, albeit lower than, the values among American adolescents (25.8 U/L for boys, 22.1 U/L for girls) [6].

The present study used the upper normal limit of ALT to estimate the prevalence of NAFLD, which revealed values of 10.8% among boys and 6.6% among girls in 2015. In contrast, use of the previous standard values (30 U/L for boys and 19 U/L for girls) generated estimated NAFLD prevalences of 6.7% among Korean boys and 5.1% among Korean girls in 2015 [10] [5, 11]. Thus, the prevalence of NAFLD in this population appears to be unexpectedly high, and we recommend aggressive management for patients who may have undetected NAFLD based on the previous standard ALT values.

The prevalence of NAFLD (elevated ALT levels (> 30 U/L)) was 3.6% in boys and 2.8% in girls from 1594 adolescents aged 10 to 19 years using the 1998 KNHANES. [12] The prevalence of NAFLD in Chinese children found out to be 9.03% with ALT thresholds > 25.8 U/L for boys > 22.1 U/L for girls using China Health and Nutrition Surveys. [13] Although there is a lack of uniformity in the data, but similar results were obtained with studies conducted in China from our study. Emma et al. pooled prevalence of NAFLD as 7.6% (95%CI: 5.5 to 10.3%) using meta-analysis and showed that it did not differ by geographical region among children and adolescents. [14, 15] Even with heterogenicity of defining NALFD across studies, it seems that the prevalence of NAFLD increase with time and has globally similar prevalence.

This study had some important limitations. First, we used elevated ALT plus elevated BMI as criteria to diagnose NAFLD. Some NAFLD patients may have normal ALT levels and it does not parallelly match the histological severity of NAFLD in children. [16] So, in order to diagnose NALFD, imaging investigations or histology confirmation should be included. However, we have not been able to confirm NAFLD using imaging investigation in this cohort study as it has not been used in every cycle of KNHANES. Therefore, it could not be included in the criteria of NAFLD in this study. However, ALT has been recommended as a screening tool for NAFLD and has previously been used for population-based epidemiological studies. [7, 8, 17] Furthermore, the upper normal limits of ALT (24.1 U/L for boys and 17.7 U/L for girls) should be validated using liver biopsies [7, 18]. Second, the KNHANES data do not include information regarding the use of hepatotoxic medication. Third, although ours is the most recent study to estimate the sex-specific upper normal limits of ALT among Korean adolescents, additional criteria should be considered when using ALT levels to estimate the prevalences of other chronic liver diseases in Korea. As we mentioned at the beginning, NAFLD should be diagnosed using liver biopsy or imaging studies. So, results from present study cannot directly be used in clinic level.

However, even with these limitation, this is first study to define upper normal ALT level of adolescents by using KHANES data. And according to our data, prevalences of adolescents NAFLD in Korea might be more serious than we used to think. As the result of this study shows normal value of ALT can varies by some circumstances, ALT laboratory thresholds used for children should be re-examined. The physicians should be aware not to underdiagnose NAFLD patient when ALT level is in normal value we used to use in clinic. However, the defining NAFLD (BMI ≥ 85th percentile plus elevated ALT) used in the study is only tools to estimate NAFLD in public populations, so this upper normal ALT results cannot directly be used in clinic. We hope these results can give clues that ALT level can be adjusted for each sex and ethnics. And ALT could be more useful tool to determine who may need more detailed medical examinations for NAFLD if ALT is adjusted specific for each ethnics and sex.

## Conclusions

The upper normal level of ALT in Korea were 24.1 U/L for boys and 17.7 U/L for girls in our study. And based on the weighting of the KNHANES design, our estimates indicate that NAFLD may be present in year 2015, approximately 282,981 adolescent boys and 159,154 adolescent girls. Thus, additional care is needed to identify Korean adolescents with undetected NAFLD and its complications. And further study is needed for evaluating sensitivity and specificity of upper normal level of ALT from this study.

## Abbreviations

BMI: Body mass index
DBP: Diastolic blood pressure
HDL: High density lipoprotein
KNHANES: Korea National Health and Nutrition Examination Survey
NAFLD: Non-alcoholic fatty liver disease
SBP: Systolic blood pressure
TG: Triglyceride
